# Fourier analysis of corneal irregular astigmatism after small-incision lenticule extraction and transepithelial photorefractive keratectomy: A comparative study

**DOI:** 10.1097/MD.0000000000037340

**Published:** 2024-03-01

**Authors:** Jiliang Ning, Lijun Zhang

**Affiliations:** a Department of Ophthalmology, The Third People’s Hospital of Dalian, Dalian, China; b Department of Ophthalmology, Dalian Municipal Eye Hospital, Dalian, China; c Liaoning Provincial Key Laboratory of Cornea and Ocular Surface Diseases, Dalian, China; d Liaoning Provincial Optometry Technology Engineering Research Center, Dalian, China.

**Keywords:** Fourier analysis, higher-order aberrations, irregular astigmatism, small-incision lenticule extraction, transepithelial photorefractive keratectomy

## Abstract

To compare changes in the spherical component, regular astigmatism, and irregular astigmatism of the anterior surface of the cornea after small-incision lenticule extraction (SMILE) and transepithelial photorefractive keratectomy (TransPRK). Fifty-six patients underwent SMILE in 56 eyes, and 68 patients underwet TransPRK in 68 eyes. The right eye was chosen to enter the group. Six months after the procedure, Scheimpflug images were acquired, and Fourier analysis of the anterior surface of patients’ corneas was performed using the Pentacam built-in software. Fourier parameters encompass various measurements such as the steepest radius of the curvature and average eccentricity of the spherical components (SphRmin and SphEcc), maximum decentration (MaxDec), central and peripheral regular astigmatism (regular astigmatism at the center [AstC] and regular astigmatism at the periphery [AstP]), and irregularity (Irr). At 6 months postoperatively, SphEcc decreased significantly (*P* < .001), MaxDec increased significantly (*P* < .001), and Irr increased insignificantly (*P* = .254) in the SMILE group. SphEcc decreased significantly (*P* < .001) and MaxDec and Irr increased significantly (*P* < .001) in the TransPRK group. TransPRK caused greater changes in SphEcc, MaxDec, and Irr on the anterior corneal surface than SMILE (*P* < .05). The amount of MaxDec-induced changes in SMILE and TransPRK was significantly correlated with the amount of higher-order aberrations and spherical aberration changes (*P* < .05). SMILE and TransPRK increase overall irregular astigmatism on the anterior surface of the cornea, more so with TransPRK, where changes in decentration are associated with with increased higher-order aberrations.

## 1. Introduction

The prevalence of myopia is on the rise globally and has emerged as a significant public health concern. It is projected that by 2050, billions of individuals will be affected by myopia.^[1]^ Refractive surgery is a significant method for treating myopia, as it has the potential to enhance the quality of life, work efficiency, and overall daily performance.^[2]^ Small-incision lenticule extraction (SMILE) and transepithelial photorefractive keratectomy (TransPRK) are 2 advanced corneal refractive surgeries with high safety, efficacy, and predictability.^[3]^ SMILE extracts the corneal stromal lens through a corneal microincision, whereas TransPRK removes the epithelium and stroma in one step, reducing flap-related complications and providing better biomechanics than femtosecond laser-assisted laser in situ keratomileusis (FS-LASIK).^[4]^ The postoperative outcomes, such as biomechanical and visual quality, of SMILE and TransPRK have been extensively studied.^[4–7]^ The spherical component and regular astigmatism were proportional to the corneal keratometric spherical degrees and astigmatism degrees. Decentration is defined as the tilt of the cornea relative to the axis of the video keratoscope. Irregularities respond to a range of optical defects that reduce visual quality.^[8]^ Decentration and higher-order irregularities can be considered irregular astigmatism because these components cannot be corrected using a spherical or astigmatic lens. Induced irregular astigmatism (decentration and irregularity) is inversely related to corrected distance visual acuity (CDVA) and contrast sensitivity after photorefractive keratectomy (PRK).^[9]^ Corneal irregularities have been shown to increase after SMILE, PRK, and FS-LASIK and may affect postoperative vision quality.^[10–12]^ Therefore, we investigated the changes in the spherical component, regular astigmatism, and irregular astigmatism (decentration and irregularity) and their correlation with the level of myopia correction and higher-order aberrations (HOAs) at 6 months after SMILE and TransPRK.

## 2. Materials and methods

### 2.1. Ethics statements

Informed consent was obtained from all the patients, and the study was conducted in accordance with the Declaration of Helsinki. The institutional review board of the Dalian Third People’s Hospital affiliated approved the protocol (approval Number: 2021-LW-007).

### 2.2. Setting, study design, and participants

This was a prospective, nonrandomized, controlled study. Patients who met the surgical indications were given the freedom to choose the surgical method based on their personal preferences. Fifty-six patients (56 eyes) underwent SMILE, and 68 patients (68 eyes) underwent TransPRK at the Third People’s Hospital of Dalian, from January 2022 to August 2022. The right eye was chosen to enter the group. All patients were between 18 and 35 years of age with equivalent spherical lens degree of −3.00 D to −6.00 D, astigmatism < 1.5 D, preoperative central corneal thickness > 500 μm, stable refractive error in the past 2 years, preoperative best-corrected visual acuity > 20/25, and preoperative discontinuation of soft corneal contact lenses for at least 1 week, rigid corneal contact lenses for 1 month, and orthokeratology lenses for 6 months. Exclusion criteria were keratoconus, suspected keratoconus, other types of keratectasia, active ocular disease, psychiatric symptoms such as anxiety and depression, diabetes mellitus, autoimmune or systemic infectious diseases, and keloid or severe dry eye.

### 2.3. Preoperative and postoperative examinations

Preoperatively, all patients underwent uncorrected distance visual acuity (UDVA) assessment, CDVA, computerized optometry, subjective optometry, noncontact intraocular pressure measurement, 6mm corneal wavefront aberration measurement by Sirus (CSO, Italy), ultrasonic corneal thickness measurement, slit-lamp microscopic eye examination, and Pentacam HR (Oculus, Wetzlar, Germany) assessment for corneal tomography. Six months postoperatively, uncorrected distance visual acuity assessment, noncontact intraocular pressure measurement, slit-lamp ophthalmoscopy, and Pentacam HR assessment for corneal tomography were performed. One instrument was used by the same physician.

### 2.4. Fourier analysis

The Fourier functions are trigonometric sine and cosine functions with increasing periodicity, and a period of 2π can be converted to a Fourier harmonic series as follows:


$$\begin{array}{l} \left(\;\varphi \;\right)=\sum \left[an*cos\left(n\;\varphi \;\right)+bn*sin\left(n\;\varphi \;\right)\right] \end{array}$$

This can be rewritten as a cosine function, including the phase-shift angle, as follows:


$$\begin{array}{l} \left(\;\varphi \;\right)=\sum \left[cn*cosn\left(\;\varphi \;+\;\alpha \;n\right)\right] \end{array}$$

Fourier analysis of circular fluctuations in corneal keratometry (K) decomposes various clinical components: N = 0, spherical equivalent (SE); N = 1, decentration; N = 2, regular astigmatism; and N > 3, irregular astigmatism.

The data were obtained from the Pentacam Fourier analysis module. Fourier analysis was performed on the K of the anterior corneal surface within an 8mm diameter around the corneal apex. The resulting parameters are listed in Table 1.

**Table 1:** Parameters obtained in Fourier analysis.

Terms	Explanation
SphRmin	Steepest radius of the curvature of spherical components
SphEcc	Average eccentricity in spherical components
MaxDec	Maximum decentration or asymmetry of astigmatic components
AstC	Central 3.5-mm regular astigmatism
AstP	Peripheral 3.5 to 7-mm regular astigmatism
Irr	Irregularity, components that cannot be corrected by a spherical lens, cylindrical lens, and prism

### 2.5. Surgical techniques and postoperative care

SMILE was performed using a Zeiss VisuMax femtosecond laser system (Carl Zeiss Meditec, Jena, Germany) for corneal cutting with set parameters: cap thickness, 120 μm; optical zone diameter (OZ), 6.0 to 6.6 mm; incision position, 120°; and size, 2 mm. The microlens was removed through the incision with micro-forceps and covered with a corneal bandage lens after surgery. Postoperatively, 0.5% levofloxacin eye drops were administered 4 times a day for 1 week. Sodium hyaluronate (0.3%) was administered 4 times a day for 3 months. Tobramycin dexamethasone drops were administered 5 times daily for 1 week, followed by 0.1% fluorometholone ophthalmic solution 4 times daily and tapered over the next month.

TransPRK was performed under SPT guidance using a Schwind Amaris 750S excimer laser system (Schwind eye-tech-solutions, Kleinostheim, Germany) with central epithelial corneal thickness set at 55 μm, peripheral corneal epithelial thickness set at 65 μm, optical zone set at 6.0 to 6.5 mm, ablation followed by 0.02% mitomycin action for 20 seconds, and postoperative coverage of corneal bandage lens. Postoperatively, 0.5% levofloxacin eye drops were administered 4 times a day for 1 week. Sodium hyaluronate (0.3%) was administered 4 times a day for 3 months. Tobramycin dexamethasone drops were administered 4 times a day for 3 days, followed by 0.1% fluorometholone drops 3 times a day, and tapered over the next 3 months.

### 2.6. Statistical analysis

The measurement data of continuous variables were assessed for normal distribution using the Shapiro–Wilk test. Data that followed a normal distribution were presented as mean ± standard deviation (SD). After setting the power of the test to 0.85, we calculated the minimum sample size for a single group to be 47 using PASS (version 15; NCSS, Inc., Kaysville, UT). The chi-square test was used to compare sex differences between the SMILE and TransPRK groups. An independent samples *t*-test was used to compare the differences in the amount of preoperative baseline information and the induced changes in steepest radius of the curvature of spherical components (SphRmin), mean eccentricity of the spherical component (SphEcc), maximum decentration (MaxDec), AstC, AstP, irregularity (Irr), and higher-order corneal aberration between the 2 groups. A paired t-test was used to compare the differences between the preoperative and postoperative parameters for SMILE and TransPRK. Correlations between SphRmin, SphEcc, MaxDec, AstC, AstP, and Irr and equivalent spherical lens degree, depth of cut, lens thickness, OZ, K value change, amount of change in total higher-order corneal aberration, amount of change in spherical aberration (SA), and amount of change in coma (CA) aberration were analyzed using Spearman rank correlation analysis. Statistical analyses were performed using SPSS software (version 22; IBM Corp., Armonk, NY). Statistical significance was set at *P* < .05.

## 3. Results

In total, 56 patients (56 eyes) underwent SMILE and 68 patients (68 eyes) underwent TransPRK. There were no significant differences in sex, age, SE, sphericity, astigmatism, best-corrected visual acuity, thinnest corneal thickness, or OZ between the SMILE and TransPRK groups before surgery. However, there was a significant difference in ablation depth (AD)/lenticule thickness (LT) between the 2 groups (*P* < .001) (Table 2).

**Table 2:** Preoperative demographic and refractive data for both groups.

Variable	SMILE	TransPRK	*P*-value
Sex, male/female	36/20	44/24	.97
Age (y)	23.79 ± 5.26	22.32 ± 5.36	.13
SE (D)	−5.27 ± 1.15	−5.11 ± 1.03	.39
Sphere (D)	−4.96 ± 1.04	−4.81 ± 1.02	.45
Astigmatism (D)	−0.64 ± 0.54	−0.58 ± 0.42	.53
CDVA (log MAR)	−0.074 ± 0.021	−0.073 ± 0.021	.93
TCT (μm)	565.36 ± 24.86	557.37 ± 24.21	.07
AD/LT (μm)	107.57 ± 16.94	90.78 ± 8.12	<.001
OZ (mm)	6.41 ± 0.33	6.42 ± 0.11	.79

Means ± SD for continuous variables.

AD = ablation depth, CDVA = corrected distance visual acuity, LT = lenticule thickness, OZ = optical zone diameter, SE = spherical equivalent, SMILE = small-incision lenticule extraction, TCT = thinnest corneal thickness, TransPRK = transepithelial photorefractive keratectomy.

After SMILE, SphRmin, MaxDec, and AstP increased significantly (*P* < .001); SphEcc and AstC decreased significantly (*P* < .02); and Irr showed a statistically nonsignificant increase (*P* = .25). After TransPRK, SphRmin, MaxDec, AstP, and Irr increased significantly (*P* < .001); SphEcc decreased significantly (*P* < .001); and AstC showed a statistically insignificant change (*P* = .9) (Table 3).

**Table 3:** Fourier parameters of the preoperative and postoperative anterior corneal surfaces in both groups.

	SMILE			TransPRK			Induced change (postop–prop) between the groups
	Preop	Postop	*P*-value	Preop	Postop	*P*-value	*P*-value
SphRmin	7.80 ± 0.26	8.06 ± 0.28	<.001	7.79 ± 0.28	8.06 ± 0.30	<.001	.97
SphEcc	0.49 ± 0.14	-0.79 ± 0.14	<.001	0.52 ± 0.10	-0.87 ± 0.18	<.001	<.001
MaxDec	0.15 ± 0.06	0.29 ± 0.11	<.001	0.13 ± 0.05	0.32 ± 0.12	<.001	.04
AstC	0.11 ± 0.05	0.09 ± 0.04	.019	0.10 ± 0.05	0.10 ± 0.05	.9	.05
AstP	0.12 ± 0.05	0.15 ± 0.08	<.001	0.10 ± 0.04	0.14 ± 0.07	<.001	.39
Irr	0.022 ± 0.025	0.026 ± 0.009	.25	0.022 ± 0.006	0.036 ± 0.016	<.001	.004

Means ± SD for continuous variables.

AstC = regular astigmatism at the center, AstP = regular astigmatism at the periphery, Irr = irregularity, MaxDec = maximum decentration, postop = postoperatively, preop = preoperatively, SMILE = small-incision lenticule extraction, SphEcc = mean eccentricity of the spherical component, SphRmin = steepest radius of the curvature of spherical components, TransPRK = transepithelial photorefractive keratectomy.

**Figure 1.**
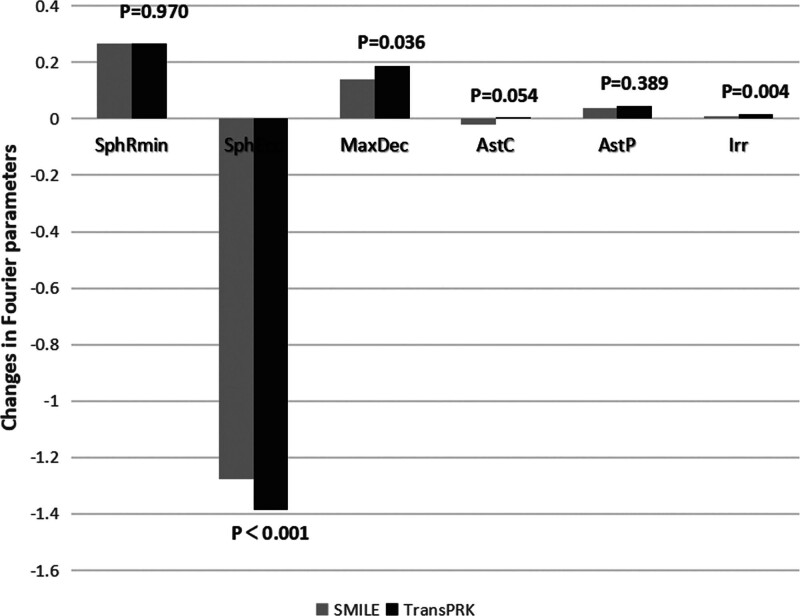
Comparison of the amount of postoperative Fourier parameter-induced changes between the 2 groups.

TransPRK caused larger changes in SphEcc, MaxDec, and Irr in the anterior corneal area than SMILE (*P* < .05), and the remaining parameters were similar between the 2 groups (Fig. 1).

After SMILE and TransPRK, total HOAs, SA, and CA increased significantly (*P* < .001). TransPRK caused larger changes in total HOAs and SA in the anterior corneal area than SMILE (*P* < .05) (Table 4).

**Table 4:** Higher-order aberrations of the preoperative and postoperative anterior corneal surfaces in both groups.

	SMILE			TransPRK			Induced change (postop–prop) between the groups
	Preop	Postop	*P*-value	Preop	Postop	*P*-value	*P*-value
HOAs (μm)	0.44 ± 0.12	0.74 ± 0.22	<.001	0.43 ± 0.12	0.96 ± 0.28	<.001	.04
SA (μm)	0.20 ± 0.08	0.47 ± 0.17	<.001	0.20 ± 0.08	0.73 ± 0.25	<.001	<.001
CA (μm)	0.27 ± 0.13	0.41 ± 0.18	<.001	0.24 ± 0.14	0.35 ± 0.18	<.001	.92

Means ± SD for continuous variables.

CA = coma, HOAs = higher-order aberrations, SA = spherical aberration, TransPRK = transepithelial photorefractive keratectomy.

Results of the correlation analysis of the Fourier parameter changes in the SMILE group are presented in Table 5. Changes in SphEcc were significantly correlated with the SE, central corneal keratometric change, LT, OZ, HOAs changes, SA changes, and CA changes (*P < *.05). Changes in MaxDec were significantly correlated with the OZ, HOAs changes, and SA changes (*P* <. 05). AstP-induced changes were significantly correlated with the SE changes, central corneal keratometric changes, LT, OZ, HOAs changes, and CA changes (*P* < .05). Irr-induced changes were significantly correlated with the central corneal keratometric changes, LT, and OZ (*P* < .05).

**Table 5:** Results of the correlation analysis of the amount of Fourier parameter-induced changes after SMILE.

	ΔSphRmin	ΔSphEcc	ΔMaxDec	ΔAstC	ΔAstP	ΔIrr
SE (D)	0.229 (.09)	-0.450 (.001)	0.043 (.76)	-0.139 (.31)	0.394 (.003)	0.225 (.09)
ΔK (D)	-0.104 (.46)	0.879 (<.001)	-0.260 (.05)	0.176 (.19)	-0.468 (<.001)	-0.387 (.003)
LT (μm)	0.160 (.24)	-0.695 (<.001)	0.163 (.23)	-0.298 (.03)	0.423 (.001)	0.369 (.005)
OZ (mm)	0.155 (.26)	0.475 (<.001)	-0.358 (.008)	0.159 (.24)	-0.263 (.06)	-0.342 (.01)
ΔHOAs (μm)	-0.206 (.16)	-0.332 (.02)	0.376 (.009)	-0.193 (.19)	0.298 (.04)	0.268 (.07)
ΔSA (μm)	-0.121 (.41)	-0.449 (.001)	0.369 (.01)	-0.167 (.26)	0.279 (.06)	0.145 (.33)
ΔCA (μm)	-0.100 (.49)	-0.319 (.02)	0.282 (.05)	-0.274 (.06)	0.322 (.03)	0.176 (.23)

AstC = regular astigmatism at the center, AstP = regular astigmatism at the periphery, CA = coma, HOAs = higher-order aberrations, Irr = irregularity, K = keratometry, LT = lenticule thickness, MaxDec = maximum decentration, OZ = optical zone diameter, SA = spherical aberration, SE = spherical equivalent, SMILE = small-incision lenticule extraction, SphEcc = mean eccentricity of the spherical component, SphRmin = steepest radius of the curvature of spherical components.

Results of the correlation analysis of changes in the Fourier parameters in the TransPRK group are shown in Table 6. Changes in SphEcc were significantly correlated with the SE, central corneal keratometric changes, AD, OZ, HOAs changes, changes in SA, and changes in CA *(P* < .05). Changes in MaxDec were significantly correlated with the SE, central corneal keratometric changes, AD, OZ, HOAs changes, and changes in SA (*P* < .01).

**Table 6:** Results of the correlation analysis of the amount of Fourier parameter-induced changes after TransPRK.

	ΔSphRmin	ΔSphEcc	ΔMaxDec	ΔAstC	ΔAstP	ΔIrr
SE(D)	−0.099 (.42)	−0.782 (<.001)	0.514 (<.001)	0.005 (.97)	0.119 (.33)	0.075 (.54)
ΔK (D)	0.110 (.37)	0.838 (<.001)	−0.478 (<.001)	−0.048 (.7)	−0.095 (.44)	-0.100 (.42)
AD (μm)	−0.008 (.95)	−0.688 (<.001)	0.457 (<.001)	0.004(0.98)	0.282 (0.02)	0.080 (0.52)
OZ (mm)	0.026 (0.83)	0.774 (<.001)	−0.494 (<.001)	0.048(0.69)	−0.157 (0.2)	-0.052 (0.67)
ΔHOAs (μm)	−0.143 (.27)	−0.661 (<.001)	0.416 (.001)	0.195 (.13)	0.126 (.33)	0.181 (.16)
ΔSA (μm)	−0.044 (.74)	−0.770 (<.001)	0.463 (<.001)	0.156 (.23)	0.066 (.66)	−0.019 (.89)
ΔCA (μm)	−0.020 (.88)	−0.255 (.05)	0.230 (.08)	0.106 (.42)	-0.146 (.26)	0.029 (.83)

AD = ablation depth, AstC = regular astigmatism at the center, AstP = regular astigmatism at the periphery, CA = coma, HOAs = higher-order aberrations, Irr = irregularity, K = keratometry, MaxDec = maximum decentration, OZ = optical zone diameter, SA = spherical aberration, SE = spherical equivalent, SphEcc = mean eccentricity of the spherical component, SphRmin = steepest radius of the curvature of spherical components, TransPRK = transepithelial photorefractive keratectomy.

## 4. Discussion

This study evaluated the changes in Fourier parameters such as the spherical component, regular astigmatism, and irregular astigmatism after SMILE and TransPRK and the associated influencing factors. Corneal refraction surgery changes the ability of the cornea to refract by reshaping its anterior surface. However, different tissue removal methods may lead to different corneal biomechanics and morphological alterations.^[10–12]^ Previous studies investigating the alteration of Fourier parameters on the anterior surface of the cornea after laser-assisted laser in situ keratomileusis and PRK showed an increase in irregular astigmatism (decentration and irregularity) on the anterior surface of the cornea postoperatively.^[9,13,14]^ Irregular astigmatism (decentration and irregularity) induced by PRK is inversely correlated with postoperative CDVA and contrast sensitivity.^[9]^

We observed a reduction in the spherical component of the cornea after SMILE and TransPRK, which correlated with a flatter postoperative cornea. TransPRK caused a greater change in the SphEcc of the anterior surface of the cornea than SMILE, suggesting that the cornea was flatter after TransPRK when correcting for the same equivalent sphere. Similarly, Zhang et al found a flatter anterior corneal surface after FS-LASIK compared to SMILE and speculated that this finding was related to the fabrication of the corneal flap and the difference in postoperative biomechanics between the 2 procedures.^[15]^ We found that the postoperative SphEcc correlated with the depth of corneal tissue removal and the diameter of the optical zone, suggesting that the postoperative spherical component of the anterior corneal surface correlated with the corneal removal profile, which may be one of the reasons for the difference in the postoperative spherical component between the 2 procedures. We also found an increase in postoperative peripheral regular astigmatism with both surgical approaches, which is consistent with the findings of Sideroudi et al^[13]^ This may be because the correction of astigmatism during SMILE and TransPRK is mainly accomplished by removing the peripheral cornea.

Previous studies have suggested that decentration originates from the video corneal image acquisition process and the central alignment strategy in the ablation zone. Decentration is calculated for the cornea relative to the axis of the video keratoscope centered on the corneal apex, which may change when the corneal apex is flattened on the anterior surface postoperatively.^[14]^ Additionally, the alignment strategy differed between the 2 procedures. TransPRK uses pupil-centric control of its strategy along with dynamic and static cyclic torsion and is adjusted by the surgeon in real time during the cutting process. In contrast, SMILE uses a corneal vertex alignment strategy and relies on subjective patient alignment, and the patient must remain immobilized under a flickering light during the scan.^[3]^ Therefore, some surgeons are unable to maintain a good central alignment strategy because of the lack of an eye movement tracking system. However, Lazaridis et al found that SMILE was superior to FS-LASIK in terms of alignment to the center, provided that both maintained good central alignment.^[16]^ This result is similar to our finding that TransPRK induced a greater centrifugal volume than SMILE did. Moreover, we verified for the first time that SMILE- and TransPRK-induced decentrations were significantly correlated with corneal aberration.

SMILE did not cause significant changes in irregularity production, but TransPRK induced an increase in irregularity, suggesting superior retinal imaging quality after SMILE.^[9]^ This is consistent with our results of greater corneal aberration after TransPRK compared to SMILE. Surprisingly, we found no significant correlation between SMILE- and TransPRK-induced irregularity and changes in corneal HOAs. We speculated that the poor repeatability of irregularities during the measurement process and the greater susceptibility to the influence of tear film stability might be related.^[8,17]^

## 5. Limitations

We have the ability to perform a well detailed analysis of the corneal tomography and evaluate the entire shape distribution of the cornea including anterior and posterior cornea together. However, we just focused on the anterior corneal curvature changes and not the total corneal changes including the posterior corneal curvature impact on the Fourier. This is due to the influence of the current pentacam version, which cannot yet derive the posterior corneal surface Fourier parameters.

## 6. Conclusions

In conclusion, SMILE and TransPRK increase irregular astigmatism on the anterior surface of the cornea, especially TransPRK, where changes in decentration are associated with increased HOAs.

## Author contributions

**Conceptualization:** Jiliang Ning.

**Data curation:** Jiliang Ning.

**Formal analysis:** Jiliang Ning.

**Funding acquisition:** Jiliang Ning.

**Investigation:** Jiliang Ning.

**Methodology:** Jiliang Ning.

**Project administration:** Jiliang Ning.

**Resources:** Jiliang Ning.

**Software:** Jiliang Ning.

**Supervision:** Lijun Zhang.

**Validation:** Jiliang Ning.

**Visualization:** Jiliang Ning.

**Writing – original draft:** Jiliang Ning.

**Writing – review & editing:** Lijun Zhang
